# The Importance of Early Diagnosis of Somatic Symptom Disorder: A Case Report

**DOI:** 10.7759/cureus.44554

**Published:** 2023-09-02

**Authors:** Atharv Sardesai, Komal N Muneshwar, Mridul Bhardwaj, Dev B Goel

**Affiliations:** 1 Medicine and Surgery, Jawaharlal Nehru Medical College, Datta Meghe Institute of Higher Education and Research, Wardha, IND; 2 Community Medicine, Jawaharlal Nehru Medical College, Datta Meghe Institute of Higher Education and Research, Wardha, IND; 3 Medicine, Jawaharlal Nehru Medical College, Datta Meghe Institute of Higher Education and Research, Wardha, IND

**Keywords:** somatic symptom disorder (ssd), comorbid depression, opioid analgesics, nsaid’s, antidepressants, somatic pain

## Abstract

A somatic symptom disorder (SSD) diagnosis is made when a person places emphasis on physical symptoms such as pain, exhaustion, or shortness of breath so much that it causes significant suffering and/or functional issues. The individual's thoughts, sentiments, and activities are an overstated reaction to such symptoms. Regardless of whether the physical symptoms are connected to a diagnosable medical condition, the person experiences symptoms and believes they are ill. When a person exhibits symptoms that satisfy the diagnostic standards of an SSD, the disease should be identified. However, due to the disorder's frequent co-occurrence, particularly with anxiety and depressive disorders, support for these concurrent diagnoses should be sought. Cognitive-behavioral therapy, mindfulness-based therapy, and medication are all examples of effective treatments for SSD. It has been demonstrated that tricyclic antidepressants or selective serotonin reuptake inhibitors (SSRIs) aid in treating symptoms.

The authors describe the case of an eight-year-old boy with complaints of abdominal pain that were unexplained by various tests. The pain lasted 10 years and was episodic (each episode lasted around 10 days; one particular episode lasted approximately six months). Multiple investigations were conducted, but no physiological reason for his symptoms was discovered. His evaluation was conducted by an interdisciplinary team that included neurologists, psychiatrists, surgeons, and doctors. The underlying cause was subsequently determined to be SSD. As people with SSD present to general practitioners and the emergency room rather than psychiatric facilities, this incident serves as a sobering reminder of the need to advocate for an accurate diagnosis of this condition.

## Introduction

In 2013, the Diagnostic and Statistical Manual of Mental Disorders, Fifth Edition (DSM-5) introduced the term somatic symptom disorder (SSD). The terms 'somatization disorder,' 'undifferentiated somatoform disorder,' 'hypochondriasis,' and 'pain disorder' were all eliminated from the manual. A patient's concern about actual symptoms without an organic explanation is this disorder’s defining trait. A psychological assessment is required to rule out co-occurring psychiatric illnesses [1]. Somatoform pain is a persistent pain for which no known physical cause exists. It is distressing, frequently interferes with daily activities, and lowers the quality of life [2]. Along with the presence of undiagnosed pain, the DSM-5 lists three factors as diagnostic indicators for SSD: 1) disproportionate and persistent thoughts about pain; 2) high levels of anxiety; and 3) spending an excessive amount of time and energy worrying about pain that interferes with daily life [3]. 

Unwary doctors or surgeons might conduct examinations or diagnostic procedures that could have iatrogenic repercussions if this sickness is not recognized. The financial strain it places on the healthcare system is likewise significant. Patients with undiagnosed pain have a poor prognosis if they continue to use medical services [1]. Adverse early experiences with caregivers may interact with a person's genetic predisposition, disrupting the maturation of the neural circuits involved and affecting regulation and interpersonal functioning, ultimately leading to the persistence of early developmental tendencies to experience somatic distress into adulthood. This is the central tenet of the developmental theory of somatic pain. 

Recent research has found that interpersonal discomfort and attachment-related consequences have neurological connections with physical pain [4]. This basic neural system may be impacted by the interplay of negative early experiences and a genetic predisposition, increasing susceptibility to somatic pain due to changing brain dynamics of physical discomfort, interpersonal distress, and emotion regulation in adulthood [5]. Antidepressants, anti-anxiety medications, and pain medications are frequently used to treat somatoform pain disorder (SPD). However, this approach often requires a long course of therapy with unpredictable results and, occasionally, serious side effects [2].

## Case presentation

An eight-year-old boy presented to the pediatrician with vague abdominal pain and was treated for constipation and worms. The pain automatically subsided after a week. Over the next year, he regularly missed school with complaints of similar abdominal pain and was recommended to see a gastrointestinal (GI) specialist. The boy gradually started describing the complaint further than just being vague.

The pain was described as shooting and colicky, generalized over the abdomen, acute in onset, severe in intensity, and not radiating. The stomach was heavily guarded. The pain aggravated severely with the ingestion of food, and was so severe that the patient used to jump up and down on the bed. It was relieved a little by stretching the abdomen. The pain was associated with constipation and occasionally vomiting and unrelated to diarrhea, nausea, fever, anorexia, jaundice, melena, or hematemesis. The pain was episodic (approximately once every six months), and each episode lasted 10 to 15 days.

These episodes occurred regularly from 2009 to 2019 (10 years). The patient's everyday life was completely disrupted during the attack, and he could not attend school or participate in any other activities. He refused to eat or pursue any interests. Sleep was heavily disturbed, and the patient was irritable. He had no history of tuberculosis, diabetes, hypertension, or asthma. There was also no significant family history. His surgical history included an appendectomy in 2016 unrelated to these episodes. Between these episodes, the patient was asymptomatic and was performing all the functions expected at that age appropriately. However, the patient was bedridden during the attacks, and his quality of life was severely hampered.

Investigations

Over the course of 10 years, various investigations were performed to rule out infectious, immune-mediated, inflammatory, congenital, nutritional, vascular, endocrine, and neoplastic etiologies. Routine blood investigations, including CBC and urine and stool analyses, were conducted every time an episode occurred. Imaging studies included six transabdominal ultrasounds, three CT scans, two contrast CT scans, and 10 X-rays. Three endoscopic explorations were done, and in 2016, an exploratory laparoscopy was performed. No abnormality was found in any of the investigations.

Treatment

The patient was treated by various specialists, including a gastroenterologist, an endocrinologist, a surgeon, and a psychiatrist. Depending on the doctor, the patient was either treated at home or hospitalized. He was hospitalized a total of five times. As the patient's father was a doctor, he was mainly treated at home with consultation from a specialist. The episodes were treated with non-steroidal anti-inflammatory drugs (NSAIDs), including ibuprofen, diclofenac, and naproxen for pain. Broad-spectrum antibiotics were prescribed empirically. Antiamoebics were also regularly prescribed. Antiemetics like ondansetron and domperidone were given for nausea. As the patient was not constipated during the episodes, stool softeners, including an enema, were provided. These medicines were given during a regular attack. However, these were not enough to counteract the pain.

An initial diagnosis of abdominal migraine was made in 2011, and treatment included IV load and oral sodium valproate. This line of treatment was continued for future episodes until 2014. The patient initially showed little symptomatic relief, but the relief subsided, and the medication was withdrawn due to excess sedation. The pain episodes stopped responding to sodium valproate after this. As the pain episode continued to be excruciating, another line of treatment in the form of opioid analgesics was deployed. Tramadol and morphine were given along with Phenergan (promethazine) for sleep. This helped with the pain, but the patient started to develop tolerance, and this line of treatment was withdrawn in 2015. After this, the pain was countered with ketamine until 2017.

During this time, the patient was in the 11th grade and started showing signs of comorbid depression. The patient showed hopelessness, loss of interest, excessive sleeping, and general discontent. A diagnosis of SSD with background depression was made subsequently, and he was started on 10 mg of escitalopram, a selective serotonin reuptake inhibitor (SSRI). Initially, there was excessive sedation; the doctors added bupropion, a norepinephrine and dopamine reuptake inhibitor (NDRI). After this, the episodes decreased in intensity and frequency. The NSAIDs were enough to ease the pain during this time. Although the attacks continued until 2020, there were only three in the span of three years (from 2017 to 2020). These were treated with NSAIDs and SSRIs, and NDRIs were continued between episodes. Since 2020, the patient has been on SSRI escitalopram and NDRI bupropion, and there has not been a single episode of somatic pain.

## Discussion

Patients suffering from SSD worry too much about getting sick. According to SSD diagnostic criteria, one must have had persistent symptoms for at least six months. Patients with SSD deem their physical symptoms overly frightening, damaging, or bothersome and frequently have pessimistic views of their health. When a person has severe SSD, their health may come to dominate their lives, shaping who they are and how they interact with others. This case differs from the traditional presentation in that the patient did not excessively worry about the illness and did not base their behavior around the disease. There were long periods between the episodes in which the patient was symptomless. This contradicts the criteria that the patient exhibits symptoms for at least six months before SSD diagnosis. Therefore, in such conditions, the diagnosis can be missed, as in our case, where it went undiagnosed for 10 years. Nevertheless, in this case, strong evidence renders a diagnosis of SSD. And so, this should be kept in mind by the physician while diagnosing such unexplained symptoms.

A person must have at least one distressing somatic symptom and excessive thoughts, feelings, or behaviors related to the somatic symptom or any health issues that are related for SSD to be diagnosed. The impacted individual's critical decline in social interaction puts the entire family's social life at risk, putting them at risk of permanent social withdrawal [6]. Developmental issues, physical and sexual abuse, cognitive and perceptual distortions, behavioral abnormalities, and difficulties with self-expression are among the psychosocial factors that may contribute to the etiology of SSD. It has been demonstrated that the emergence of physical and psychological issues is linked to life transitions. Family conflict is another known risk factor for its etiology. As a result of their somatic symptoms, people with depressive disorders typically experience GI symptoms like bloating, diarrhea, nausea, and vomiting. Headaches, chest pain, joint pain, and back pain are among the agonizing effects. Atypical movement, sensory loss, weakness, and paralysis are all neurological symptoms. Somatization and stress are closely linked [7]. A comprehensive treatment plan tailored to each patient is required. Somatic symptom disorders typically last for a long time. However, only 10% to 30% of people experience deterioration, while 50% to 75% experience improvement. A decrease in physical symptoms and an increase in baseline functionality are positive prognostic indicators.

The postgraduate medical curriculum lacks sufficient instruction in diagnosing and treating this condition. This subject needs scientific investigation. A bio-psycho-social framework, rehabilitative models, and behavioral therapy should be used in assessment and treatment. To address specific symptoms and restrictions, methods like distraction, muscle relaxation for headaches, graduated physical exercise for sore muscles and fatigue, and practical management of pseudo-seizures can all be used [8]. Tricyclic antidepressants (TCA), specific SSRIs, specific serotonin and noradrenalin reuptake inhibitors (SNRIs), abnormal antipsychotics, and natural cures are the five primary pharmacological classes that have been taken into consideration. The data indicate that each of the five groups is successful in treating a variety of ailments. All antidepressant classes appear to effectively treat somatoform disorders and related conditions. The SSRIs appear to be more effective than other antidepressants when pain is the primary symptom. There was some evidence, albeit scant and of poor quality, that the various antidepressant classes were all equally effective [9].

Tricyclic antidepressants had a remarkable track record of performance and were more likely to be effective than SSRIs. Amitriptyline is the tricyclic that has received the most research, and it is thought to be useful for at least one of the following conditions: a tender point score (based on the number and severity of tender points) and functional symptoms, pain, stiffness in the morning, general improvement, improved sleep, and fatigue. Prozac (fluoxetine) showed promise among the SSRIs tested for pain, function, overall well-being, sleep, morning stiffness, and tender spots [9]. The biopsychosocial approach is a crucial step in determining the contributing and maintaining factors for children and adolescents with SSD, ensuring that they benefit only from interventions that are supported by scientific evidence. Cognitive-behavioral therapy is the treatment of choice for these patients. To maintain the patient's age-appropriate activity level, the therapy ought to concentrate on lessening psychosocial stresses, health anxiety, and criticism from the patient's family. Additionally, it should aim to improve communication and coping skills [10]. The efficacy of drugs other than antidepressants, a deeper comparison of antidepressants, and longer follow-ups (the longest follow-up was just 12 weeks) should all be the subject of future studies of the highest caliber. Additionally, outcomes such as symptom intensity, sadness, or anxiety, as well as functional impairment or dysfunctional behavior and cognitions, should be taken into consideration in the studies [11].

## Conclusions

In the context of primary care, one of the most common categories of patient concerns is SSD, which affects a large portion of the general population in the context of psychiatric disorders. A persistent somatic illness affects a significant portion of people who first experience acute somatic symptoms. These conditions may manifest during childhood, adolescence, or adulthood. The prevalence of SSD in girls is estimated to be higher than in boys. A significant deterioration in health status is linked to the disease. More than two standard deviations below population norms regarding deteriorated health status are anticipated for many people with severe SSDs. Beyond the essential physical deterioration, a patient faces various psychological and social difficulties. 

When searched in PubMed, the term 'somatic symptom disorder' yields 5111 results. Out of these, only 117 are systematic reviews, while 627 are review articles. A mere 289 cases are seen as clinical trials. The reporting of this disease has increased substantially in the last decade, with nearly 3000 articles, of which 1800 were published in the last five years. Even with this increasing trend in publishing articles related to the disorder, there is still an urgent need for wider reporting to better understand the disease and enable more doctors to help more patients. 

As the case described shows, the longer the duration of the undiagnosed period, the higher the toll on the patient. Extended hospitalization puts the patient at risk for infections, and pursuing various lines of treatment is expensive. Hence, early diagnosis is of the essence in such cases. Not only is it a pillar of primary prevention, but it also helps plan the treatment for such patients. An early diagnosis enables people to make informed decisions regarding their care, finances, and options. Early detection and treatment are essential to preventing, managing, and treating SSDs.
